# Methamphetamine-Induced Blood Pressure Sensitization Correlates with Morphological Alterations within A1/C1 Catecholamine Neurons

**DOI:** 10.3390/ijms251910282

**Published:** 2024-09-24

**Authors:** Carla Letizia Busceti, Domenico Bucci, Antonio Damato, Massimiliano De Lucia, Eleonora Venturini, Michela Ferrucci, Gloria Lazzeri, Stefano Puglisi-Allegra, Mariarosaria Scioli, Albino Carrizzo, Ferdinando Nicoletti, Carmine Vecchione, Francesco Fornai

**Affiliations:** 1 Istituto di Ricovero e Cura a Carattere Scientifico (IRCCS) Neuromed, 86077 Pozzilli, Italy; carla.busceti@neuromed.it (C.L.B.); domenico.bucci@neuromed.it (D.B.); antonio.damato85@gmail.com (A.D.); massidelucia.m@libero.it (M.D.L.); eleonora.venturini94@libero.it (E.V.); stefano.puglisiallegra@neuromed.it (S.P.-A.); stabulario@neuromed.it (M.S.); albino.carrizzo@unisa.it (A.C.); ferdinandonicoletti@hotmail.com (F.N.); 2 Department of Translational Research and New Technologies in Medicine and Surgery, University of Pisa, 56126 Pisa, Italy; michela.ferrucci@unipi.it (M.F.); gloria.lazzeri@unipi.it (G.L.); 3 Department of Medicine, Surgery and Dentistry, “Scuola Medica Salernitana” University of Salerno, 84081 Baronissi, Italy; 4 Department of Physiology and Pharmacology, University Sapienza, 00185 Roma, Italy

**Keywords:** methamphetamine sensitization, catecholamines, ventrolateral medulla, blood pressure, vascular reactivity, free radicals, p62, HSP70, phospho-cJun, GAD, α-synuclein

## Abstract

Methamphetamine (METH) is a drug of abuse, which induces behavioral sensitization following repeated doses. Since METH alters blood pressure, in the present study we assessed whether systolic and diastolic blood pressure (SBP and DBP, respectively) are sensitized as well. In this context, we investigated whether alterations develop within A1/C1 neurons in the vasomotor center. C57Bl/6J male mice were administered METH (5 mg/kg, daily for 5 consecutive days). Blood pressure was measured by tail-cuff plethysmography. We found a sensitized response both to SBP and DBP, along with a significant decrease of catecholamine neurons within A1/C1 (both in the rostral and caudal ventrolateral medulla), while no changes were detected in glutamic acid decarboxylase. The decrease of catecholamine neurons was neither associated with the appearance of degeneration-related marker Fluoro-Jade B nor with altered expression of α-synuclein. Rather, it was associated with reduced free radicals and phospho-cJun and increased heat shock protein-70 and p62/sequestosome within A1/C1 cells. Blood pressure sensitization was not associated with altered arterial reactivity. These data indicate that reiterated METH administration may increase blood pressure persistently and may predispose to an increased cardiovascular response to METH. These data may be relevant to explain cardiovascular events following METH administration and stressful conditions.

## 1. Introduction

Methamphetamine (METH) is a widely abused psychostimulant that produces behavioral sensitization, which is related to the onset of addiction. 

Apart from addiction, METH induces structural damage associated with altered autophagy, mitochondrial dysfunction, short-lasting massive increase in oxidative species and free radicals [1,2,3,4,5,6].

METH-induced structural damage in vivo has been mainly characterized within sub-cortical brain regions, mostly the meso-striatal dopamine (DA) system. In fact, METH destroys meso-striatal DA terminals, which is concomitant with a decrease in striatal DA levels [7,8,9] and a loss of a few DA cell bodies within the substantia nigra *pars compacta* [10,11]. Therefore, METH treatment may be used as a model of parkinsonism [12,13]. Nonetheless, it is well documented that METH administration induces systemic alterations, including cardiovascular, respiratory and metabolic functions. Hassan and collaborators [14] demonstrated that a single dose of METH significantly alters the control of the sympathetic nervous system, affecting cardiovascular and metabolic activity. It is remarkable that these changes may be magnified following repeated METH administration. In fact, we recently published that METH produces a sensitization in the elevation of systolic blood pressure (SBP). This occurs for METH doses which are higher than those required to induce locomotor sensitization [15]. Blood pressure sensitization occurs following reiterated doses of METH and persists at prolonged time intervals even during METH withdrawal. This suggests the onset of a long-lasting, self-sustaining, arterial hypertension. METH-induced sensitization was previously characterized concerning locomotor (ambulatory) activity, which occurs following repeated injection of METH. The occurrence of vascular sensitization represents a novelty in METH research. In fact, these recent findings only analyzed systolic blood pressure (SBP) in the absence of any morphological correlation, and diastolic blood pressure (DBP) was not analyzed. Moreover, no data exist concerning which anatomical area or protein alteration may be involved. 

Therefore, the present experimental study was designed to investigate whether, along with sensitized SBP, DBP was also similarly modified. Moreover, in the search for anatomical correlates for these effects, a number of immunohistochemistry experiments were carried out in the lower brainstem where neurons responsible for blood pressure regulation are placed [15]. In addition, considering that persistent alterations of NE release are likely to occur within orthosympathetic nerve endings, the reactivity of the vascular arterial wall was assessed in response to a various stimulus, such as phenylephrine and acethylcholine (ACh).

In the lower brainstem, tyrosine-hydroxylase (TH)-containing catecholamine neurons were stereologically counted within vasomotor nuclei of the medullary reticular formation. Data about catecholamine cell number in these brainstem areas were correlated with values of blood pressure. Moreover, in these areas, further morphological markers were assessed, such as dihydroethidium (DHE) to stain free radicals, occurrence of heat shock protein 70 (HSP70), phospho-c-jun, p62/sequestosome, α-synuclein and glutamic acid decarboxylase (GAD), which were analyzed either alone or in combination with TH to assess potential co-localization within A1/C1 regions of the lower brainstem, where a relevant center, which is a kernel to control blood pressure, is placed.

The present analysis is focused on those small though key neuronal aggregates in the medulla, since these nuclei play a pivotal role in blood pressure regulation. Thus, the present study wishes to establish whether catecholamine nuclei within A1/C1 corresponding to the caudal ventrolateral medulla and rostral ventrolateral medulla (CVLM and RVLM, respectively) own a higher vulnerability compared with rostral catecholamine nuclei. Moreover, we assessed whether a change in these neurons may be associated with both SBP and DBP sensitization following METH administration. 

## 2. Results

### 2.1. METH-Induced SBP Sensitization

**Figure 1:**
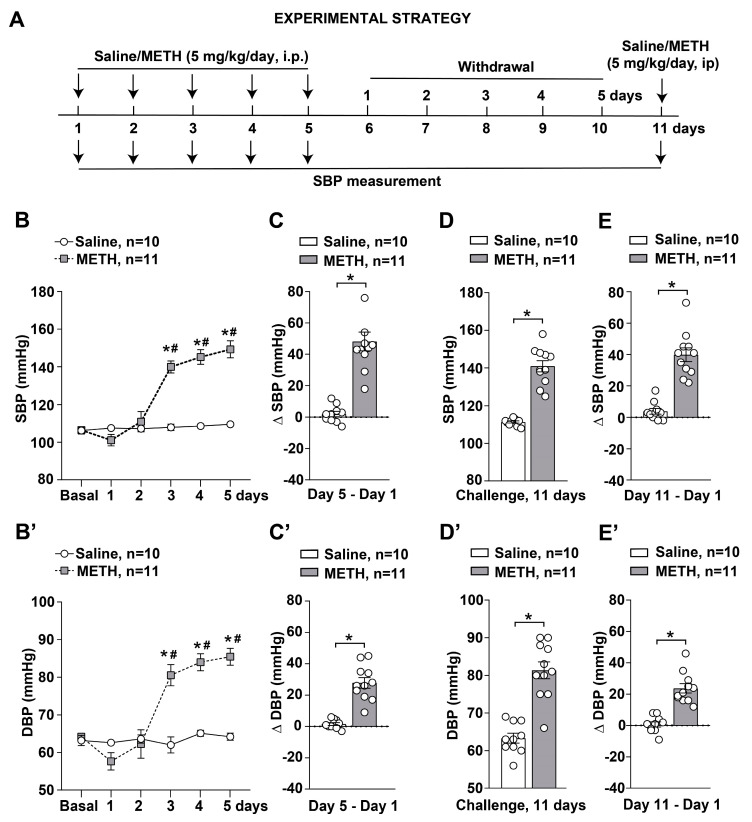
Methamphetamine (METH) induces SBP and DBP sensitization in mice. (**A**) Diagram showing the protocol used for monitoring SBP and DBP values in mice subjected to daily intraperitoneal (i.p.) injection of saline or METH (5 mg/kg) for 5 days and then re-challenged with saline or METH following 6 days of withdrawal on day 11. SBP and DBP were monitored by the tail-cuff plethysmography before treatments (Basal) and 30 min after each injection with saline or METH. SBP and DBP values monitored in mice treated with saline or METH for 5 days are shown in (**B**,**B’**)**,** respectively. Values are the means ± S.E.M. *p* < 0.05, (*) vs. saline; (#) vs. Basal, day 1 and day 2 (two-way ANOVA for repeated measures with Bonferroni’s test). Difference in SBP values (Δ SBP) and DBP values (Δ DBP) monitored at 30 min after saline or METH injections between days 5 and day 1 (Day 5–Day 1) is shown in (**C**,**C’**), respectively. Values are the means ± S.E.M. * *p* < 0.05 (unpaired two-tailed Student’s *t*-test). SBP and DBP values monitored in mice at 30 min after the challenge with saline or METH performed following 6 days of withdrawal on day 11 are shown in (**D**,**D’**), respectively. Values are the means ± S.E.M. * *p* < 0.05 (unpaired two-tailed Student’s *t*-test). Difference in SBP values (Δ SBP) and DBP values (Δ DBP) monitored at 30 min after saline or METH injections between days 11 and day 1 (Day 11–Day 1) are shown in (**E**,**E’**), respectively. Values are the means ± S.E.M. * *p* < 0.05 (unpaired two-tailed Student’s *t*-test).

Here, we show that repeated METH administration (5 mg/kg, i.p. for 5 days) triggers an increase in both SBP and DBP. These effects occur as a typical sensitization process, which follows repeated injections (Figure 1B,B’,C,C’). Differing from behavioral sensitization, blood pressure-induced sensitization occurs following higher METH doses, since 1 mg/kg and 2 mg/kg are not effective, while also producing behavioral sensitization [15]. In pilot experiments, we also tried doses exceeding these three published protocols, ranging between 0.1 mg/kg and 10 mg/kg. We found the best behavioral sensitization for 1 mg/kg and 2 mg/kg, while the best BP sensitization occurs following 5 mg/kg. It is difficult to translate this dosing to humans due to the fast metabolism which takes place in rodents. Nonetheless, it has been demonstrated that behavioral sensitization and increase in blood pressure does take place in humans following a range of doses, which are therapeutically used to treat attention deficit hyperactivity disorder (ADHD) [16]. Thus, there is not a clear-cut separation between therapeutic and harmful METH doses, which contributes to the debate whether METH administration should be carried out for ADHD and eating disorders. 

The hypertensive response to METH is long-lasting and self-sustaining during METH withdrawal, as observed following the challenge carried out after 5 days of METH withdrawal (Figure 1D,D’,E,E’).

Since METH acts in the periphery at the level of the arterial wall, one may argue that repeated METH administration produces a sensitization due to altered arterial reactivity.

**Figure 2:**
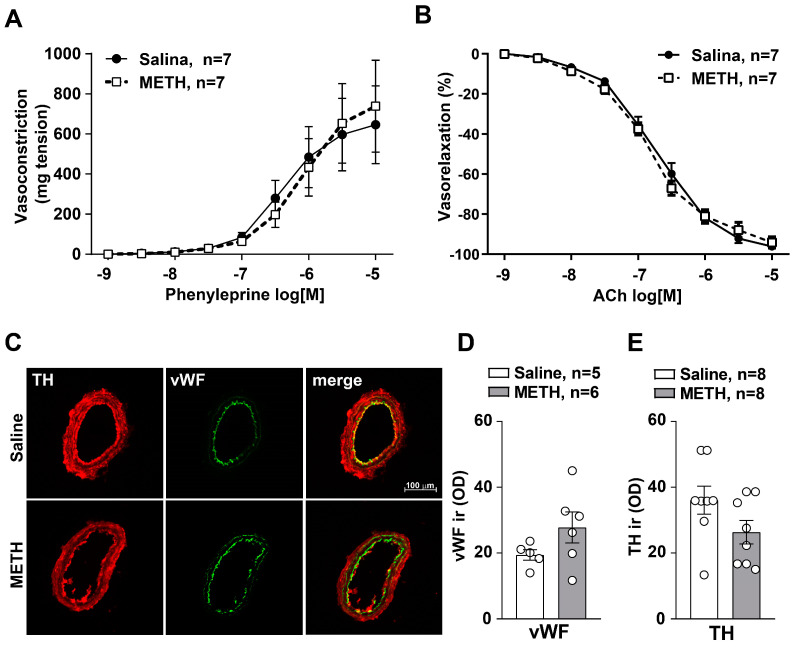
Reiterated exposure to METH in mice does not lead to mesenteric arteries’ endothelial disfunction. Mesenteric arteries were isolated from mice subjected to repeated injections of saline or METH (5 mg/kg, i.p., for 5 days) and then sacrificed 60 min after the challenge, carried out on day 11. Phenylephrine-induced vasoconstriction and acetylcholine (ACh)-induced vasorelaxation in mesenteric arteries isolated from saline or METH-treated mice are shown in (**A**,**B**), respectively. Values are means ± S.E.M. (**C**) Double fluorescent staining for the catecholaminergic marker tyrosine hydroxylase (TH) and the endothelial marker von Willebrand factor (vWF) in mesenteric arteries isolated from mice treated with saline or METH and sacrificed 60 min following the challenge with saline or METH performed following 6 days of withdrawal on day 11. Densitometric quantification of the vWF or TH immunoreactivity (ir) is shown in (**D**,**E**), respectively. Values of optical density (OD) are means ± S.E.M.

Therefore, we examined whether the hypertensive effects induced in response to repeated treatments with METH could be due to an exaggerated arterial response to the vasoconstrictor activity induced by norepinephrine (NE). Alternatively, a suppression of the arterial response to ACh may lead to altered regulation by the vascular endothelium. Therefore, we measured vascular reactivity in resistance vessels (mesenteric arteries) isolated from saline or METH-injected mice. Treatment with METH did not affect either phenylephrine-mediated vasoconstriction (Figure 2A) or ACh-mediated vasodilation (Figure 2B). This indicates that no change in vascular reactivity occurred. Nonetheless, since METH increases NE release, one may still consider that repeated stimulation of presynaptic orthosympathetic nerve endings at the level of the arterial wall may be responsible for the onset of SBP sensitization. This is likely to be the case due to increased activity promoted by medullary vasomotor centers. Still, no morphological alterations were detected concerning both orthosympathetic and parasympathetic nerve endings within the arterial wall when immunohistochemistry was carried out. In fact, double immunofluorescence analysis of TH expression co-localized with von Willebrand factor (vWF) did not provide a significant difference when tested within sections of mesenteric arteries (Figure 2C–E). This indicates that METH-induced SBP and DBP sensitization was not related to changes within the arterial wall (neither functional nor anatomical) in either the muscle or endothelial layers. It is important to emphasize that these results were obtained within resistance vessels which are responsible for regulating vascular resistance, thereby affecting, according to the Poiselle’s law, blood pressure. 

### 2.2. METH Sensitization Is Concomitant with Reduced Catecholamine Cell Density in A1/C1 of the Rostral and Caudal Ventrolateral Medulla (RVLM and CVLM)

**Figure 3:**
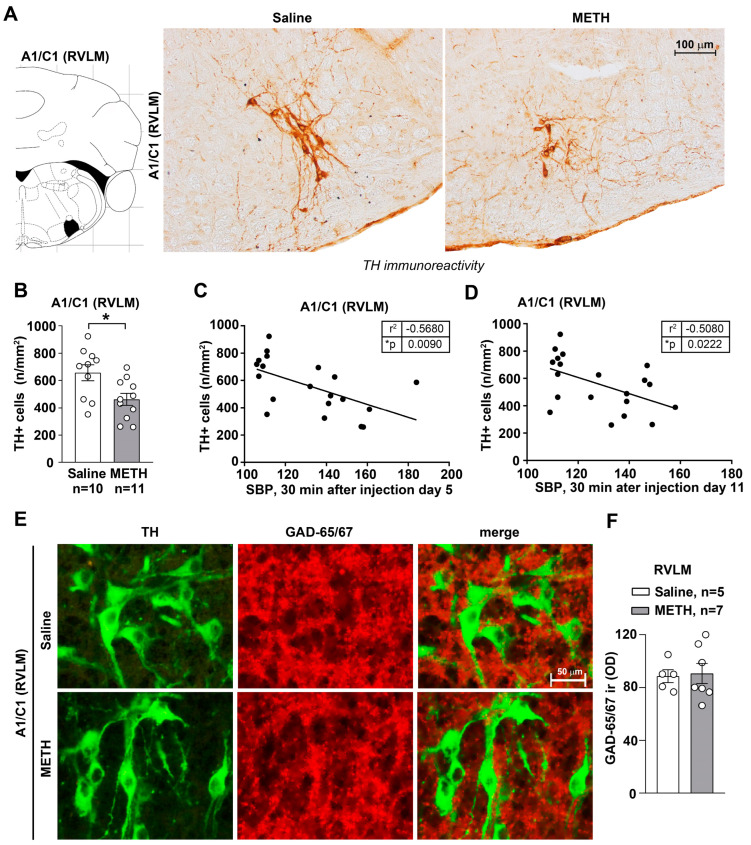
Within the RVLM, repeated METH administration reduces TH immunopositive cells without affecting GAD immunopositive cells. Immunohistochemical analysis for the catecholamine marker TH was carried out from dissected brains of mice subjected to repeated injections of saline or METH (5 mg/kg, i.p., for 5 days) and then sacrificed 60 min after the METH/saline challenge, which was carried out on day 11. Representative images of TH-positive neurons within A1/C1 in the RVLM of mice treated with saline or METH are shown in (**A**). The graph in (**B**) shows TH-positive cell density (n/mm^2^) counted in saline- and METH-treated mice. Values are the means ± S.E.M. * *p* < 0.05 (unpaired two-tailed Student’s *t*-test). The correlation analyses between TH-positive cell density within A1/C1 in the RVLM and SBP are shown in (**C**,**D**), respectively. These correlation analyses were carried out considering SBP values that were measured at 30 min following the fifth saline/METH injection or following the METH/saline challenge. * *p* < 0.05, Pearson correlation test. (**E**) Representative images of double immunostaining for TH and GAD in the RVLM of mice following repeated injections of saline or METH (5 mg/kg, i.p., for 5 days). These mice were sacrificed 60 min following the METH-/saline challenge, carried out on day 11. The densitometry (OD: Optical Density) of GAD65/67 immunoreactivity (ir) is shown in (**F**). Values are the means ± S.E.M. * *p* < 0.05 (two-tailed Mann-Whitney test).

**Figure 4:**
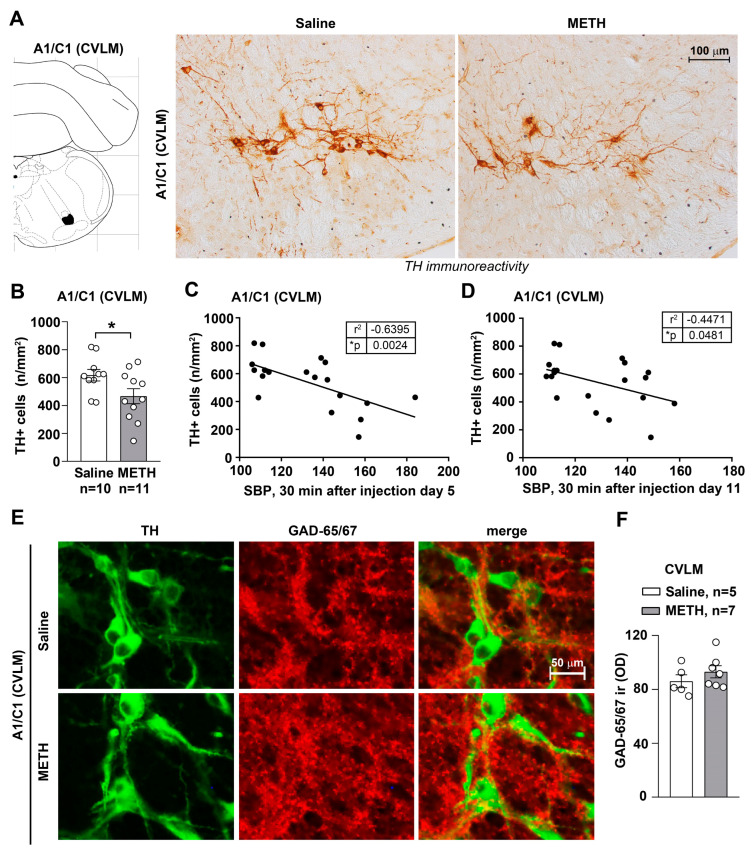
Within CVLM, repeated METH administration reduces TH immunopositive cells without affecting GAD immunopositive cells. Immunohistochemical analysis for TH was performed in dissected brains of mice subjected to repeated injections of saline or METH (5 mg/kg, i.p., for 5 days) and then sacrificed 60 min after the challenge, carried out on day 11. Representative images or TH-positive neurons in A1/C1 within the CVLM of mice treated with saline or METH are shown in (**A**)**.** The graph in (**B**) shows the TH-positive cell density (n/mm^2^) counted in saline- and METH-treated mice. Values are the means ± S.E.M. * *p* < 0.05 (unpaired two-tailed Student’s *t*-test). The correlation analysis between values of TH-positive cell density in A1/C1 of the CVLM and the respective SBP values monitored 30 min after the fifth repeated saline/METH injection or following the challenge carried out on day 11 is shown in (**C**,**D**), respectively. * *p* < 0.05, Pearson correlation test. (**E**) Representative images of double immunostaining for TH and GAD in the CVLM of mice following repeated injections of saline or METH (5 mg/kg, i.p., for 5 days). These mice were sacrificed 60 min following the METH/saline challenge, carried out on day 11. The densitometry (OD: Optical Density) of GAD65/67 immunoreactivity (ir) is shown in (**F**). Values are the means ± S.E.M. * *p* < 0.05 (two-tailed Mann-Whitney test).

**Figure 5:**
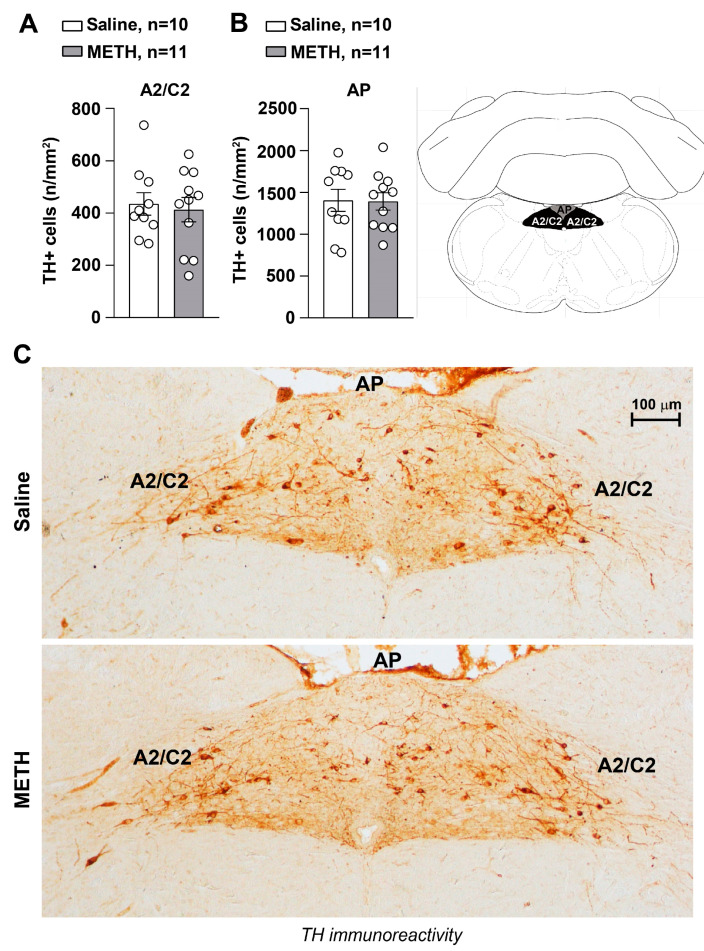
Within A2/C2 and AP, METH does not decrease catecholamine neurons. Immunohistochemical analysis for TH was performed in dissected brains of mice following repeated METH administration (5 mg/kg, i.p., for 5 days). Mice were sacrificed 60 min following the METH challenge, which was carried out on day 11. TH-positive cell density (n/mm^2^) counted in saline- and METH-treated mice is shown in (**A**,**B**), respectively. Values are the means ± S.E.M. Representative images or TH-positive neurons in A2/C2 and AP of mice treated with saline or METH are shown in (**C**).

In the lack of functional and anatomical persistent changes intrinsic within the arterial wall, the study moved to brainstem centers involved in the control of blood pressure, which potentially also modify peripheral NE release. No morphological effects were detected in a recent manuscript concerning the higher brainstem (pons and mesencephalon) of blood pressure sensitized mice [15]. Therefore, in the present work, we extended the analysis to lower brainstem nuclei where medullary centers involved in cardiovascular control are placed [17]. In fact, our data published a few weeks ago showed that repeated treatments with METH did not damage the catecholamine meso-accumbal system or noradrenergic neurons in the Locus Coeruleus [15]. Instead, when immunohistochemical analysis of TH immunoreactive neurons was carried out in these lower brainstem areas, repeated METH administration led to reduced catecholamine cell density in A1/C1 both within the RVLM (Figure 3A,B) and CVLM (Figure 4A,B). In contrast, no changes in catecholamine neuronal density were detected within either the dorsomedial nucleus of ala cinerea A2/C2 (Figure 5A,C) or within the area postrema AP (Figure 5B,C). This indicates that METH-induced morphological alterations are associated with a selective decrease of A1/C1 catecholamine neurons. It is remarkable that GAD-immunostaining was not modified within either TH-containing neurons or in the surrounding cells placed within the RVLM (Figure 3E,F) and CVLM (Figure 4E,F).

In order to analyse such an association, a correlation analysis was carried out between the number of TH-positive cells in A1/C1 of the RVLM and values of SBP and DBP. A similar analysis was carried out for the CVLM. Values of SBP and DBP were taken at 30 min after treatment with saline or METH, both at day 5 and day 11. A negative correlation was measured between the number of TH-positive cells and both SBP and DBP at day 5 and day 11, both in the RVLM (Figure 3C,D) and the CVLM (Figure 4C,D). These data indicate that reduced TH-positive cells found in A1/C1 following repeated METH administration are concomitant with the onset of a sensitized response to SBP and DBP. These morphological changes may partly underly the neurobiology of METH-induced SBP and DBP sensitization. Molecular mechanisms remain unknown and call for a number of future investigations.

### 2.3. METH Treatment Did Not Induce Neurodegeneration/Neurotoxicity within the RVLM and CVLM 

**Figure 6:**
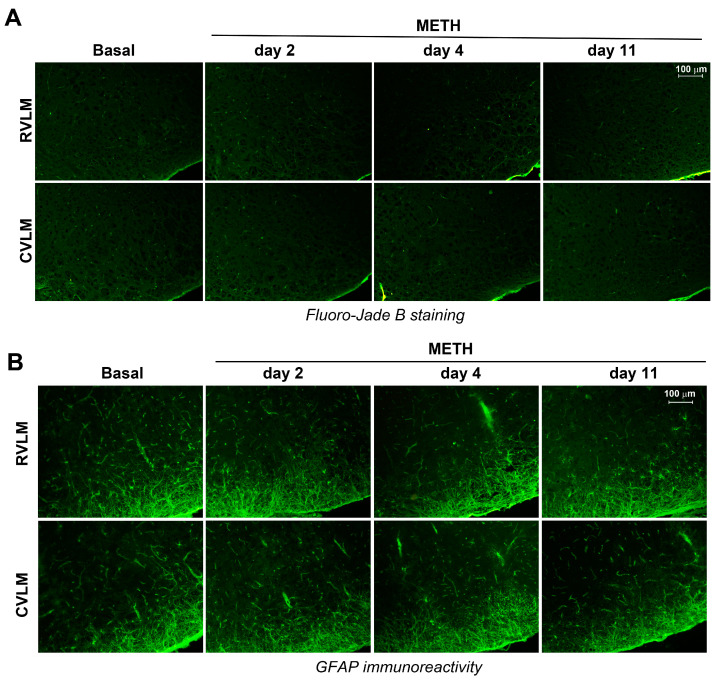
Lack of neuronal loss in the RVLM and CVLM of mice treated with METH. (**A**) Fluoro-Jade B staining was carried out within the RVLM and CVLM of mice treated with METH and sacrificed at the following different time points: (i) naïve mice (Basal); (ii) 24 h after a single treatment with METH (5 mg/kg, i.p.) (day 2); (iii) 24 h after the last injection in mice subjected to repeated injection with METH (5 mg/kg, i.p., for 3 days) (day 4); (iv) 60 min the challenge with METH (5 mg/kg, i.p.) performed following 6 days of withdrawal after a repeated treatment with METH (5 mg/kg, i.p., for 5 days) (day 11). (**B**) Immunohistochemical analysis for the glial fibrillary acidic protein (GFAP) in the RVLM and CVLM of mice subjected to a single or repeated injection of METH and sacrificed at different time intervals (see above).

To establish whether a decrease in TH-positive cell number was due to degenerative phenomena, a histological assessment of neurodegeneration using Fluoro-Jade B histofluorescence was carried out. In detail, the potential occurrence of Fluoro-Jade B positive neurons within the RVLM and CVLM of mice following METH administration was assessed. The times chosen for the analysis were the following: (i) naïve mice (Basal); (ii) 24 h after a single METH injection (day 2); (iii) 24 h after the last treatment in mice subjected to three consecutive METH injections (day 4); (iv) 60 min after the challenge with METH (5 mg/kg, i.p.) carried out following 6 days of withdrawal after repeated METH injections for 5 consecutive days (day 11). We could not find any degenerating cells within either the RVLM or CVLM at any time interval (Figure 6A). 

The occurrence of reactive gliosis in the same time window was assessed by performing an immunohistochemical analysis for the glial fibrillary acidic protein (GFAP) as a marker of astroglial activation and gliosis, which often occurs concomitantly with neurodegeneration. Again, no reactive gliosis was documented, neither in the RVLM nor in the CVLM of mice treated with METH at all time points examined (Figure 6B).

**Figure 7:**
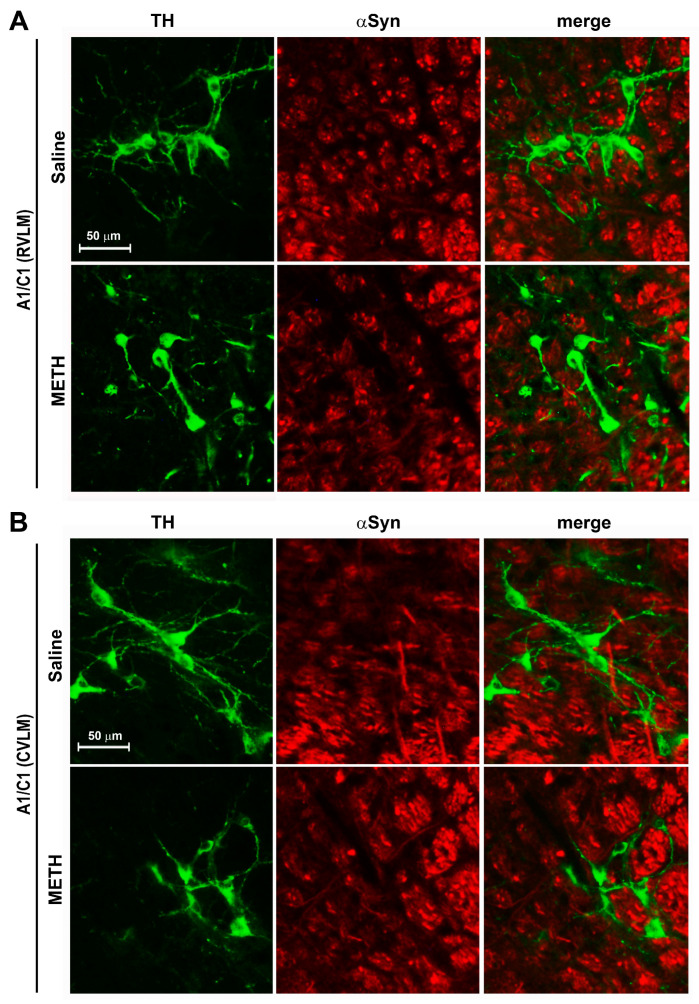
METH does not increase αSyn within A1/C1 neurons of the RVLM and CVLM. Double immunofluorescence for the catecholamine marker TH and αSyn was performed in the RVLM and CVLM of mice following repeated injections of saline or METH (5 mg/kg, i.p., for 5 days) and sacrificed 60 min after the METH challenge, which was carried out on day 11. Representative images of the double immunostaining for TH and αSyn in A1/C1 within the RVLM and CVLM of mice treated with saline or METH are shown in (**A**,**B**), respectively.

As a further observation, which is consistent with current beliefs in catacholamine degeneration, concerns the staining of catecholamine cells with the deleterious protein alpha-synuclein (αSyn). Therefore, immunohistochemical analysis of the expression level of αSyn as a marker of neurodegeneration was carried out within TH medullary neurons following repeated METH exposure [18]. With this aim, double fluorescent staining for TH and αSyn was carried out within the RVLM and CVLM from mice treated with saline or METH (5 mg/kg, i.p.) according to the sensitization protocol. No increase in αSyn expression was found within TH-positive cells of the RVLM (Figure 7A) and CVLM (Figure 7B).

Taken together, these data suggest that a METH-induced decrease of TH-positive cells within the RVLM and CVLM is not associated with neurodegeneration.

### 2.4. METH Reduces Free Radicals within A1/C1 of the RVLM and CVLM 

**Figure 8:**
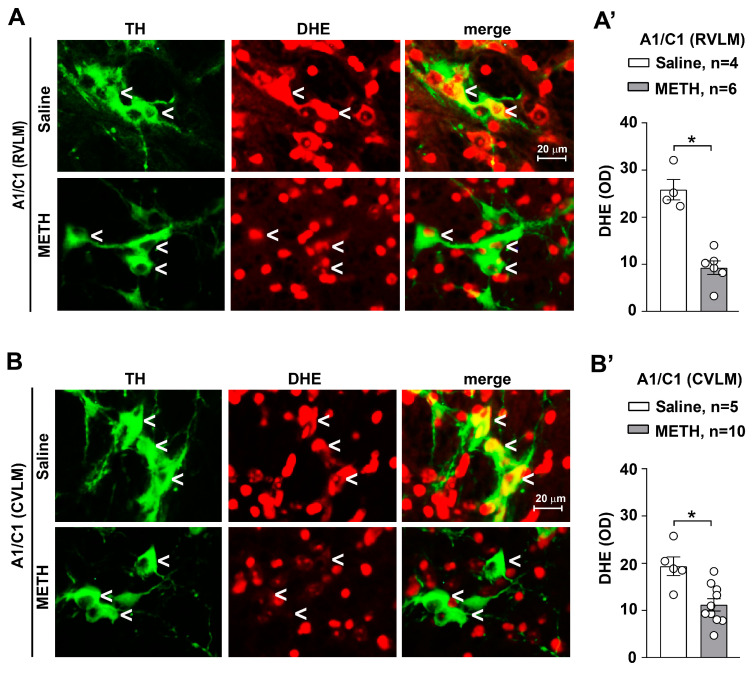
Repeated administrations of METH lead to intracellular reduction of free radicals in catecholamine A1/C1 neurons of the RVLM and CVLM. Double fluorescent analysis for TH-immunoreactivity for the catecholamine marker TH and αSyn was performed in the RVLM and CVLM of mice subjected to repeated injections of saline or METH (5 mg/kg, i.p., for 5 days) and sacrificed 60 min after the challenge carried out, on day 11. Representative images of the double immunostaining for TH and αSyn in A1/C1 within the RVLM and CVLM of mice treated with saline or METH are shown in (**A**,**B**), respectively. The respective densitometric values of DHE stain optical density (OD) are shown in (**A’**,**B’**), respectively. Values are the means ± S.E.M. * *p* < 0.05 (two-tailed Mann–Whitney test).

In the search for sub-cellular changes within medullary TH neurons of the RVLM and CVLM, intracellular levels of free radicals were assessed. With this aim, we performed a double fluorescent analysis for TH and DHE staining (for analysis of superoxide production) within the RVLM and CVLM of mice subjected to repeated injections of METH. Following the time interval when withdrawal takes place, free radicals were suppressed within A1/C1 catecholamine cells of the RVLM (Figure 8A) and CVLM (Figure 8B) compared with controls. These findings may be due to adaptive responses leading to strengthened antioxidant responses. 

### 2.5. METH Increases Heat Shock Protein 70 (HSP70) within A1/C1 of Both the RVLM and CVLM 

**Figure 9:**
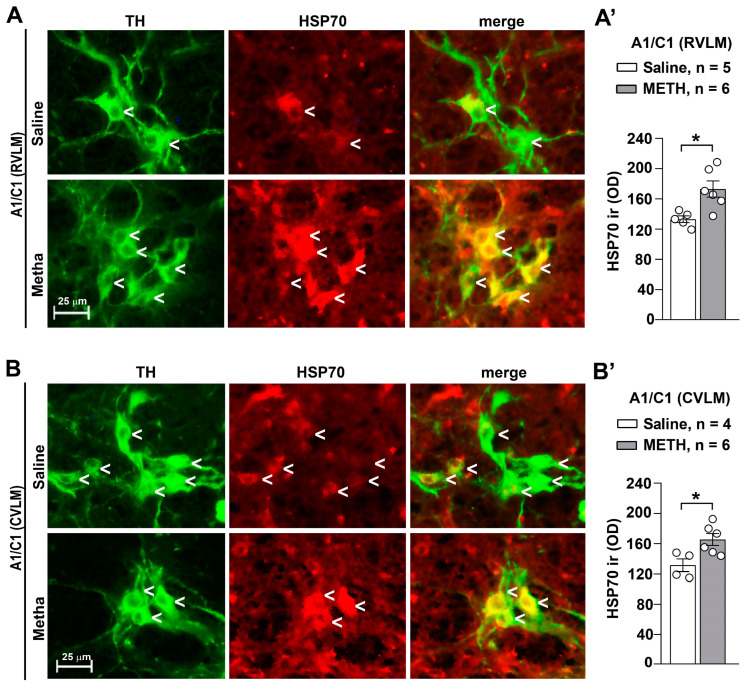
Repeated administrations of METH increase HSP70 within A1/C1 neurons. Representative images of the double immunostaining for TH and HSP70 in A1/C1 within the RVLM and CVLM of mice treated with saline or METH are shown in (**A**,**B**), respectively. The densitometry values (OD: Optical Density) of HSP70 immunoreactivity (ir) are shown in (**A’**,**B’**), respectively. Values are the means ± S.E.M. * *p* < 0.05 (two-tailed Mann–Whitney test).

In the search for further molecules that may contribute to counteract oxidative stress as adaptive responses, we assessed HSP70. Indeed, following METH administration at blood pressure sensitizing dosing, an increase in HSP70 was detected by immunofluorescence within the RVLM (Figure 9A) and CVLM (Figure 9B). It is remarkable that such an increase was merging with TH immunostaining, which indicates that the augmentation of HSP70 is quite specific for TH-immunopositive cells in both areas, as reported in the graphs of Figure 9A’ (RVLM) and 9B’ (CVLM). This suggests that HSP70 may work to compensate as a chaperone protein to decrease oxidizing species and free radicals in the cell. 

### 2.6. METH Increases Sequestosome/p62 within A1/C1 of Both the RVLM and CVLM 

**Figure 10:**
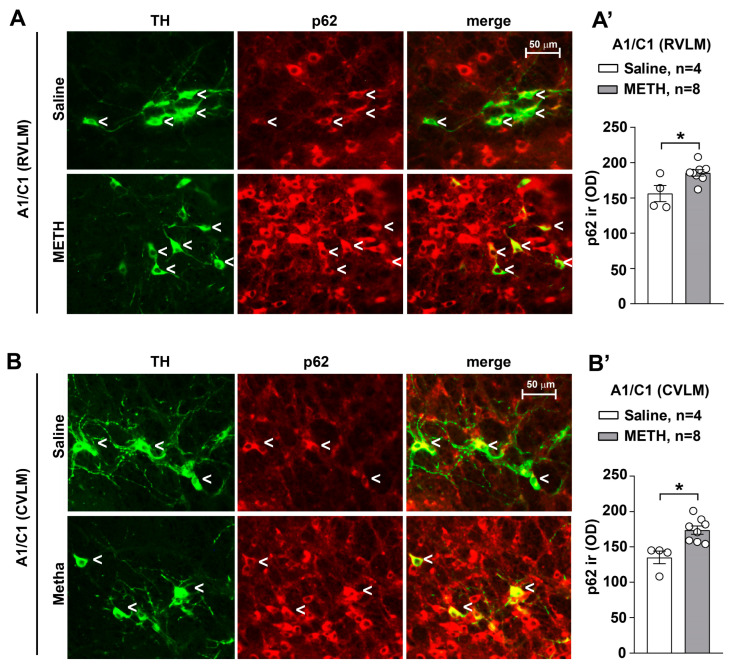
Following METH administration, p62 increases within A1/C1 neurons of the RVLM and CVLM. Double immunofluorescent analysis for TH and p62 was performed in the RVLM and CVLM of mice some days following METH administration. Representative images of double immunostaining for TH and p62 in A1/C1 within the RVLM and CVLM of mice treated with saline or METH are shown in (**A**,**B**), respectively. The values of p62 are shown in (**A’**,**B’**), respectively. Values are the means ± S.E.M. * *p* < 0.05 (two-tailed Mann–Whitney test).

To streghten the hypothesis about potential adaptive changes within these neurons, we assessed the amount of p62 or sequestosome (SQSTM1), which is a transcriptional target of the intracellular mechanisms involved in the modulation of the cellular antioxidant responses such as Keap1-NRF1 (Kelch-like) [19,20,21,22]. Consistently, a dual fluorescent expression analysis for TH and p62 showed increased expression of the p62 factor in TH-positive neurons of the CVLM/RVLM of mice following METH treatment compared with controls (Figure 10).

### 2.7. Phospho-cJun (p-cJun) Decreases Following a METH-Sensitization Protocol Leading to Increased Blood Pressure

**Figure 11:**
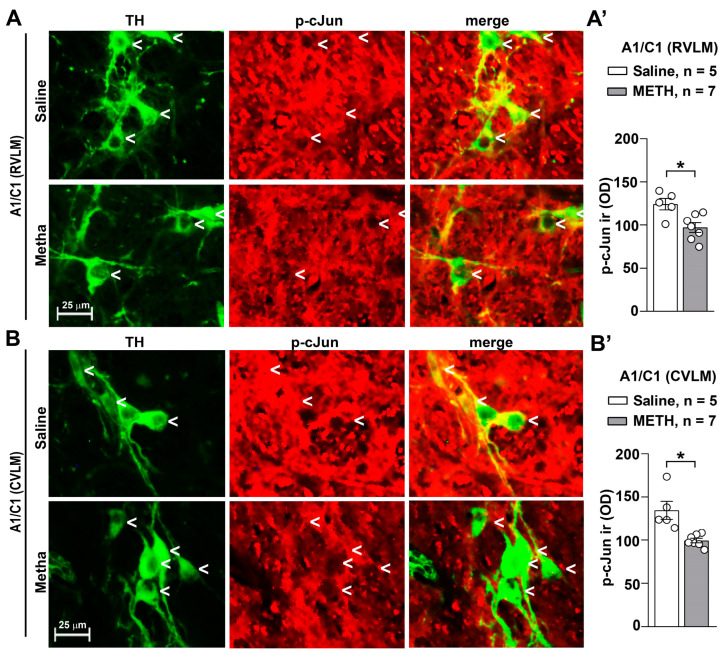
Repeated administrations of METH lead to intracellular increased p-cJun expression in catecholamine A1/C1 neurons of the RVLM and CVLM. Double immunofluorescent analysis for TH and p-cJun was performed in the RVLM and CVLM of mice subjected to repeated injections of saline or METH (5 mg/kg, i.p., for 5 days) and sacrificed 60 min after the challenge, carried out on day 11. Representative images of the double immunostaining for TH and p-cJun in A1/C1 within the RVLM and CVLM of mice treated with saline or METH are shown in (**A**,**B**), respectively. The respective densitometric values (OD: Optical Density) of p-cJun intracellular immunoreactivity (ir) are shown in (**A’**,**B’**), respectively. Values are the means ± S.E.M. * *p* < 0.05 (two-tailed Mann–Whitney test).

The double immunostaining for TH and p-cJun (Figure 11) indicates that METH administration, when carried out for repeated days and withdrawn for additional days, leads to a decrease in transcriptional activation of the enzyme TH. In fact, p-cJun acts as a factor which sustains TH transcription. Thus, a decreased in p-cJun is expected to decrease the transcription of TH. This is in line with the decrease in free radicals we measured within the same TH-positive neurons of both the RVLM (Figure 11A) and CVLM (Figure 11B). In fact, free radicals activate c-jun through its phosphorylation operated by MAP-kinases. Thus, a decrease in free radicals necessarily leads to a suppression of c-jun phosphorylation, which leads to decrease levels of p-cJun, as shown here. 

**Figure 12:**
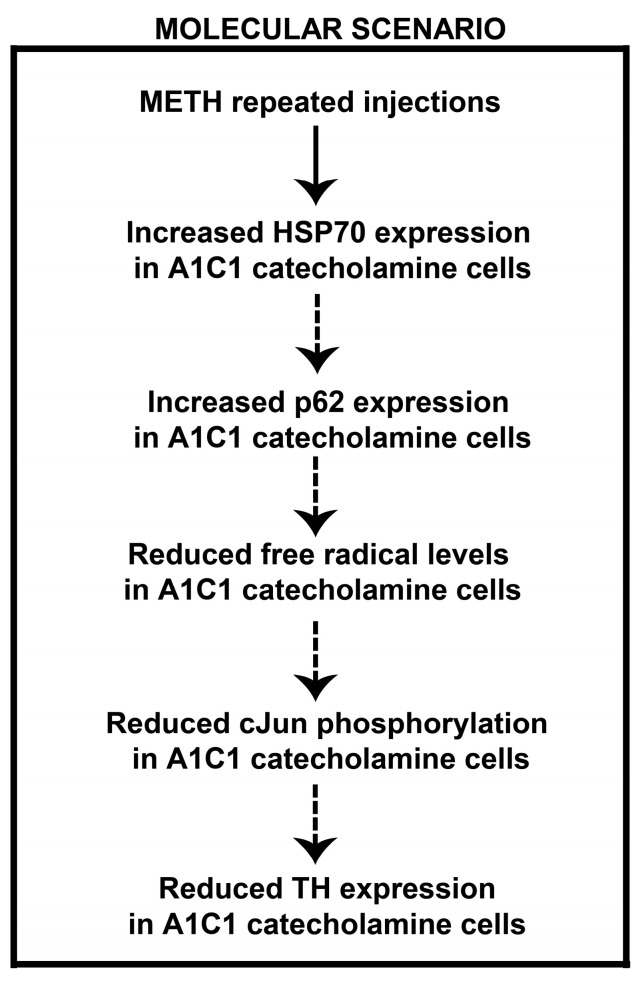
Diagram showing a possible molecular scenario occurring in response to repeated METH exposure in A1/C1 catecholamine neurons. Our findings demonstrate that, following repeated METH exposure, increased expression of HSP70 and p62 occurs in A1/C1 catecholamine neurons in the RVLM and CVLM. The consequent antioxidant effect may be responsible for the observed reduced intracellular levels of free radicals in the same cells. This may underlie a reduced phosphorylation of the transcriptional factor cJun and a downregulated expression of TH with a consequent phenotypic shift of the A1/C1 catecholamine cells within the RVLM and CVLM. The expression of p-cJun, which is also a marker of neuronal activity, is suppressed in the withdrawal phase following METH administration. This remains a quite general marker, which should be implemented by dedicated electrophysiological studies to be carried out in specific future research projects. Dotted arrows indicate that a causal relationship between each event is not demonstrated. This hypothesis warrants further investigation.

These findings confirm the occurrence of METH-induced SBP sensitization, which extends to DBP, which is sensitized similarly by the same doses and timing of METH. These effects on blood pressure are concomitant with a decrease in TH neurons of the lower medulla. Such a finding may be due to counteractive, adaptive responses based on p62 and HSP70 overexpression, which lead to decreased free radical levels. In fact, free radicals, by acting on MAPK, sustain TH transcription, possibly via c-Jun phosphorylation (Figure 12). Contrarywise, decreases of free radicals are expected to reduce the amount of TH, potentially decreasing the number of TH-positive neurons. A plethora of mechanisms may be involved in the association between sensitization of SBP and DBP and a decrease in TH neurons. Nonetheless, these measurements may disclose some key proteins, which are likely to play a mechanistic role in sustaining these phenomena. The fact evidenced by the present study remains anchored to the association between blood pressure sensitization and decreased TH neurons in A1/C1 areas (Figure 12). The expression of p-cJun, which is also a marker of neuronal activity, is suppressed in the withdrawal phase following METH administration. This remains a quite general marker, which should be implemented by dedicated electrophysiological studies to be carried out in specific future research projects. 

## 3. Discussion

The present study indicates that reiterated METH administration at the dose of 5 mg/kg produces a sensitization of both SBP and DBP. This is associated with reduced TH-immunoreactive neurons of A1/C1 within the RVLM and CVLM where the kernel for blood pressure regulation occurs. In detail, morphometric analysis of cell density of A1/C1 catecholamine neurons within the RVLM and CVLM, the dorsomedial nucleus of ala cinerea A2/C2 and the AP was carried out in brain samples disseted from mice repeatedly injected with saline or METH. We found a reduction in A1/C1 catecholamine cell density in response to treatment with METH as compared to control mice treated with saline. This cell reduction was selectively found in A1/C1 catecholamine nuclei, while no changes were detected within the other examined brainstem nuclei. In addition, we recently demonstrated that repeated daily administration of METH in mice, at the same dosing regimen used in this study, did not induce changes in nigro-striatal and meso-accumbal dopaminergic innervation, loss in dopaminergic neurons within the substantia nigra *pars compacta* or ventral tegmental area or noradrenergic neurons within the pontine nucleus of the Locus Coerulus [15]. 

Thus, a quite selective and specific involvement of the A1/C1 neurons of the RVLM and CVLM following repeated METH dosing is likely to participate in SBP and DBP sensitization. A direct molecular relationship between the occurrence of SBP/DBP sensitization and the decrease A1/C1 cell density reduction in response to METH exposure cannot be established. Even the induction of c-fos within this region does not differ from controls [15]. Specific research projects aimed at assessing neuronal activity through electrophysiology may address directly such an issue. Nonetheless, we documented that the nature of the decrease in TH neurons is unlikely to depend on degenerative or toxic events since Fluoro-Jade B and αSyn were not modified in these neurons. In fact, the expression levels of α-Syn and its intracellular accumulation typically underlies neurodegeneration [18,23]. Increased levels of α-Syn in the brain are considered a pathological phenotype and characterize neuropathological conditions defined as synucleinopathies. In line with neurotoxic effects attributed to METH, an intense increase in intracellular levels of α-Syn was documented at the level of dopaminergic neurons of the *pars compacta* of the substantia nigra of mice subjected to a protocol of acute administration of toxic doses of METH (5 mg/kg × 3 administrations carried out at 2 h intervals) [23]. In our experimental conditions involving repeated administration METH for 5 days, no increase in intracellular α-Syn levels was observed in A1/C1 catecholamine neurons of the RVLM and CVLM areas. Overall, these findings suggest that the reduction in the number of A1/C1 catecholamine cells observed within the RVLM/CVLM of mice treated with METH is unlikely to depend on neurodegenerative/toxic effects induced by the drug, but rather they may be produced by phenotypic modifications. In contrast, the free radical marker DHE was decreased along with the protein p-cJun. Contrarywise, the proteins HSP70 and p62, which possess a significant role in protecting against free radicals and oxidation, were elevated. Thus, it is likely that the sudden METH-induced increase in free radicals and oxidative species [13] may generate an adaptive response to counteract such an increase, where HSP70 and p62 are participating. This may lead to the subsequent decrease in the amount of free radicals and p-cJun. In fact, free radicals are thought to activate MAPK, which in turn phopsphorylates cJun (p-cJun). Such a phosphorylated form is likely to produce the transcriptional activation of TH. This would explain why a decrease in free radicals and decreased c-Jun activation causes a decrease in TH expressing neurons within A1/C1. Of course, this chain of events remains a hypothesis to be further tested by a number of experimental approaches. The solid evidence provided by the manuscript concerns the significant increase in SBP and DBP produced by METH at dosing which does not operate a locomotor sensitization. This effect is definitely correlated with a significant decrease in the number of TH-positive neurons within A1/C1. The chain of events which binds these phenomena remains to be fully investigated beyond the small pieces of connected evidence provided here. Nonetheless, the strength of the present findings is relevant since sensitization and an increase in blood pressure does take place in humans following a range of doses, which are therapeutically used to treat attention deficit hyperactivity disorder (ADHD) [16]. Thus, there is not a clear-cut separation between therapeutic and harmful METH doses, which contributes to the debate on whether METH should be used as a therapeutic agent.

Still, in keeping with potential mechanisms, the present work positively rules out a change in vascular reactivity following METH exposure. In fact, morphological and functional investigations on mesenteric arteries isolated from mice subjected to repeated injections of saline or METH failed to document intrinsic arterial changes. It is well known that structural and functional alterations in mesenteric vascular function contribute to the hypertensive trait [24,25,26]. However, in these experimental conditions, no alterations in phenylephrine-mediated vasoconstriction or ACh-mediated vasorelaxation in isolated mesenteric arteries was documented. This suggests that METH exposure does not lead to a long-term altered vasoreactivity of peripheral resistance vessels. Moreover, no alteration was documented for catecholamine fibers innervating the mesenteric arteries in response to METH sensitization. Still, one would expect that the activity of autonomic NE-containing axon terminals is modified within METH-sensitized mice. This is likely to occur as a mere increase in neurotransmitter release under the effects of sensitized hindbrain vasomotor centers. Consistently, with a fundamental role played by the A1/C1 nucleus in cardiovascular control, we found an inverse correlation between SBP and DBP values measured in response to repeated treatment with METH and cell number at the level of the catecholamine nuclei of the CVLM and RVLM region of the brainstem. 

A1/C1 neurons within the RVLM region play a fundamental and well-documented role in the control of blood pressure and the regulation of the sympathetic tone. Neurons from A1/C1 directly project to the cells of the intermediate-lateral columns of the spinal cord [17,27,28,29] and establish monosynaptic connections with the dorsal motor nucleus of the vagus (DMV) [30,31,32,33,34]. The role of catecholamine neurons within A1/C1 is rather modulatory on reticulo-spinal projecting neurons. In fact, unlike most brainstem neurons, C1 neurons contain mRNA for vesicular glutamate transporter type 2 (VGLUT2) [35] and appear to be excitatory and utilize glutamate as a neurotransmitter [36,37]. Most C1 neurons (90%) do not express membrane transporters for monoamines [38], and there is no direct evidence that these cells are able to release catecholamines [39]. However, they express all the enzymes necessary for neurotransmitter synthesis and vesicular storage [40,41], and then it is plausible that they are able to release catecholamines (including adrenaline). Furthermore, C1 neurons express α2-adrenergic receptors on their soma [42,43]. These autoreceptors can mediate autocrine self-inhibition [44,45]. Thus, the reduced expression of TH in C1 cells of the RVLM region in response to METH treatment could represent a reduced autocrine self-inhibition capacity of the cells of the vasomotor center resulting in increased neuronal activation in response to pressor stimuli.

Concerning the CVLM, this is a brainstem region rich in GABAergic neurons that project directly to the RVLM vasomotor nucleus [46,47]. GABAergic neurons of the CVLM are activated by afferents from baroreceptors and exert their effects on the autonomic nervous system by inhibiting the activity of pre-sympathetic neurons of the RVLM. A1/C1 catecholamine cells within the CVLM are a cell population distinct from the GABAergic component responsible for the inhibitory control over the neurons of the RVLM region [46]. Most of A1/C1 cells within the CVLM region project to the hypothalamus, and their activity is inhibited by increases in blood pressure [46,48,49,50,51,52]. These NE projections from the CVLM to the hypothalamic nuclei are primarily excitatory [53,54,55,56,57,58,59] and play a critical role in regulating cardiovascular homeostasis by activating the release of vasopressin in the bloodstream [48,58,60,61,62,63,64,65,66]. Circulating vasopressin levels modulate the baroreceptor reflex through a receptor action carried out at the level of the circumventricular organs. 

### Appendix: A Working Hypothesis

An important role in this context is played by the area postrema, one of the main circumventricular organs, which, thanks to its anatomical location, is intimately connected with the solitary tract nucleus, the main center of integration of sensory inputs deriving from the arterial baroreceptors. Neurons in the area postrema also receive direct excitatory inputs from baroreceptors, suggesting an integrative role of neuronal and humoral inputs in the control of the baroreceptor reflex and blood pressure [67]. The angiotensin (AT1A) and vasopressin (V1) receptors are, in fact, abundantly expressed in this area [68,69,70]. Indeed, vasopressin excites and angiotensin inhibits the activity of neurons in the nucleus of the solitary tract when these peptides are applied to the area postrema of rabbits [71]. Angiotensin II acts on the baroreceptor reflex by inhibiting it and resetting the threshold activation value to higher blood pressure values, thus favoring a potentially hypertensive condition. Conversely, vasopressin increases the sensitivity of the reflex by shifting its activation point towards lower blood pressure values [72,73,74]. In vivo studies and in vitro electrophysiology experiments have demonstrated the angiotensin II-induced excitatory responses in 50% of rat area postrema neurons [75]. Vasopressin also affects the neuronal excitability in the area postrema, inhibiting the firing rate in 45% of neurons in the area postrema of rats and increasing it in 38% of them [76]. As a working hypothesis which needs to be tested in future experiments, one may consider that the reduction in the number of cells expressing a catecholaminergic phenotype in the CVLM region in response to repeated treatment with METH may be causally involved in the induction of blood pressure increase due to a reduced release of circulating vasopressin. Further investigations will allow us to examine the validity of this hypothesis.

Searching for a better characterization of cellular events underlying the reduction in catecholamine cell density in A1/C1 within the RVLM and CVLM, we performed histological and immunohistochemical analyses aimed to indagate the occurrence of METH-induced neurodegeneration or neurotoxicity in these brainstem areas. We did not find any evidence of neurodegeneration or development of reactive gliosis or even an increase in free radicals after repeated METH treatments either in a time window covering a period including the early phase after the first METH injection or the long-term phase following a 6-day period of withdrawal after repeated METH injections. These data indicate that the METH-induced reduced catecholamine cell density in the A1/C1 within the RVLM and CVLM regions are unlikely to be produced by neurodegenerative events. 

Searching for a biochemical mechanism underlying METH-induced A1/C1 catecholamine reduction in the RVLM/CVLM, we performed a histological assessment of intracellular levels of free radicals. We were surprised to observe a reduced intracellular level of free radicals in A1/C1 catecholamine cells in the RVLM and CVLM regions of mice subjected to repeated treatment with METH as compared to control saline-injected mice. This finding suggests the onset of adaptive responses that lead to a strengthening of the antioxidant response. Remarkably, it is known that the intracellular production of free radicals in response to the activation of angiotensin receptors leads to the intracellular activation of the MAPK kinase (mitogen-activated protein kinase) with consequent activation of gene transcription factors, such as the activating protein-1 (AP1), responsible for the induction of expression of the TH enzyme [77]. Therefore, it can be hypothesized that the reduced level of free radicals induced by adaptive responses to METH may underlie a reduced expression of the TH enzyme with a consequent reduction of the catecholamine phenotype in A1/C1 cells of the RVLM and CVLM regions (Figure 12). 

An intracellular mechanism involved in the modulation of the cellular antioxidant responses is represented by the Keap1-NRF1 (Kelch-like) intracellular pathway. NRF2 is a transcription factor that activates the transcription of genes that contain antioxidant response regulatory elements (ARE) within the promoter [19,20,21,22]. Among these target genes, there is the p62/SQSTM1 factor or sequestosome, which is part of the intracellular degradation mechanism known as the autophagic process [78,79,80,81,82,83]. Remarkably, dual fluorescence expression analysis showed an upregulated expression of the p62/SQSTM1 factor in catecholamine neurons of the CVLM/RVLM of mice subjected to METH-induced SBP sensitization. This rises the intriguing possibility that drug exposure may be responsible for adaptive responses mediated by the p62-induced antioxidant pathway (Figure 12). Further studies will allow us to examine the causal role of this pathway in the cellular plasticity underlying the reduction of catecholamine neurons observed in the mouse brainstem in response to METH treatment.

## 4. Materials and Methods

### 4.1. Materials

METH hydrochloride was purchased from Sigma Aldrich (St. Louis, MO, USA).

### 4.2. Animals and Housing

C57Bl/6J mice were originally provided by Charles River (Charles River, Calco, LC, Italy), and bred at IRCCS Neuromed according to the FELASA recommendations for health monitoring of mouse colonies housed in conventionally controlled animal facilities. Two-month-old C57Bl/6J male mice were used for the study. Only male mice were used for experiments to avoid any potential interference of ovarian steroids on METH-induced neuronal plasticity and cerebrovascular function. Mice were housed under controlled conditions (temperature, 22 °C; humidity, 40%) with a 12 h light/dark cycle and food and water ad libitum. The study was approved by the Italian Ministry of Health (authorization #1065/2016-PR). All efforts were made to minimize animal suffering and reduce the number of animals used. 

### 4.3. Experimental Strategies

Sensitization to METH was induced in mice using a protocol consisting of daily administration for 5 consecutive days as previously reported [15]. Mice were injected intraperitoneally (i.p.) with saline or METH (5 mg/kg) daily for 5 consecutive days (day 1–day 5). After 6 days of withdrawal (day 11), mice were re-challenged with either saline or METH. SBP and DBP were measured by a tail-cuff system 30 min after each administration of METH/saline (Figure 1A). All mice were sacrificed on day 11 immediately after SBP and DBP monitoring performed 30 min after the last METH/saline injection. Mesenteric arteries isolated from sacrificed mice were used for vascular reactivity studies and immunohistochemical analysis. Dissected brains were used for histological and immunohistochemical analysis.

Separate groups of mice were treated with saline or METH and used for assessment of neurodegeneration/neurotoxicity in a selected time window covering the early period following the treatments. With this aim, mice (5 mice per group) were sacrificed at the following time points: (i) naïve mice; (ii) 24 h after a single METH (5 mg/kg, i.p.) injection; (iii) 24 h after the last treatment in mice subjected to three consecutive METH injections (5 mg/kg, i.p., for 3 days); (iv) 60 min after the challenge with METH performed after 6 days of withdrawal following repeated METH injections (5 mg/kg, i.p., for 5 days).

### 4.4. Blood Pressure Measurement

SBP and DBP were measured in mice by tail-cuff plethysmography as previously described [84,85]. Briefly, animals were placed in a holder on a temperature-controlled platform (kept at 37 °C), and recordings were performed in steady-state conditions. SBP and DBP values were averaged from at least 3 consecutive measurements.

### 4.5. Vascular Reactivity 

Vascular reactivity studies were performed in mesenteric arteries isolated from mice subjected to repeated treatment with saline or METH (5 mg/kg, i.p., for 5 days) and sacrificed 60 min after the last challenge with saline or METH carried out on day 11 at 6 days of withdrawal following repeated injections with saline or METH (5 mg/kg, i.p., for 5 days). Second-order branches of the mesenteric arterial tree were removed, and vascular studies were performed as described previously [86]. Briefly, after isolation, adventitial fat was carefully removed, and arteries were cut into segments. Subsequently, vessels were placed in a pressure myograph system (Danish Myo Technology, Hinnerup, Denmark) filled with Krebs solution maintained at a pH of 7.4 at 37 °C. 

Vasoconstriction was assessed with increasing doses of phenylephrine (from 10^−9^ M to 10^−5^ M), and the corresponding values are reported as milligrams (mg) of tension registered on wire myograph. Endothelium-dependent relaxations were assessed by measuring the dilatory response of mesenteric arteries to cumulative increasing concentrations of ACh (from 10^−9^ M to 10^−5^ M) in vessels pre-contracted with phenylephrine at a dose necessary to obtain a similar level of pre-contraction in each vessel (80% of initial KCl-evoked contraction). 

### 4.6. Immunohistochemical Analysis in Medullary Catecholamine Nuclei

Dissected brains were fixed overnight at 4 °C in Carnoy’s solution (60% ethanol, 10% acetic acid, 30% chloroform) and then embedded in paraffin. Fifteen μm serial sections sampled along the whole rostro-caudal extent of the medullary reticular formation were cut by a rotative microtome (Leica RM2245, Wetzlar, Germany; code: #RM 2245) and used for histological or immunohistochemical analysis. 

Immunoperoxidase staining for TH was performed on sections deparaffinized by immersion in xylene (30 min) and rehydrated by serial dilutions of ethanol. After that, sections were subjected to antigen retrieval by incubation in 10 mM, pH 6.0 citrate buffer heated in a microwave for 20 min. Sections were then treated at RT for 15 min with 0.1% triton X-100 (Sigma Aldrich MI, Italy; code: #93443) in phosphate buffer solution (PBS) for permeabilization and incubated for 10 min at room temperature (RT) in 3% hydrogen peroxide to block endogenous peroxidase activity. Blocking was performed by incubation for 60 min at RT in PBS/6% normal horse serum (NHS; Vector Laboratories; Burlingame, CA; code: #S-2000). Afterward, slices were incubated overnight at 4 °C with the primary antibody mouse monoclonal anti-TH (Sigma Aldrich; code: #T1299, 1:100 in PBS/3% NHS) and then for 10 min at RT with a secondary biotinylated antibody horse anti-mouse IgG (Vector Laboratories; code: #BA-2000, 1:400 in PBS, 10 min at RT). Finally, slices were incubated for 5 min at RT with horseradish peroxidase conjugated streptavidin (Vector Laboratories; code: #SA-5004, 1:100 in PBS) and then for 3 min with 3,3-diaminobenzidine tetrachloride (Sigma Aldrich; code: #D4293-50set).

Immunofluorescent staining for GFAP was performed on sections deparaffinized by immersion in xylene (30 min) and rehydrated by serial dilutions of ethanol. After that, sections were treated at RT for 15 min with 0.1% triton X-100 in PBS for permeabilization and then blocked by incubation for 60 min at RT in PBS/6% NHS. Afterward, slices were incubated overnight at 4 °C with the primary antibody mouse monoclonal anti-GFAP (Sigma-Aldrich; code: #SAB4501162, 1:300 in PBS/3% NHS) and then with a secondary biotinylated antibody horse anti-mouse IgG (1:400 in PBS, 10 min at RT; Vector Laboratories, Burlingame, CA, USA; code: #BA-2000). After the incubation with the secondary antibody, all sections were incubated for 60 min at RT with Alexa-Fluor-488 conjugated streptavidin (1:200 in PBS, Life Technologies, Eugene, OR, USA; code: #S32354).

Additional deparaffinized sections were used for double florescence analyses. To do that, sections were subjected to antigen retrieval (10 mM, pH 6.0 citrate buffer heated in a microwave for 20 min), permeabilization in triton X-100 (0.1% in PBS for 15 min at RT), and non-specific binding sites blockade with 10% normal goat serum (NGS, Vector Laboratories; code: #S-1000) in 0.1% triton X-100 in PBS for 2 h at RT. After that, sections were incubated overnight at 4 °C with a mixture containing the following primary antibodies: mouse monoclonal anti-TH (Sigma Aldrich; code #T1299, 1:100) and a rabbit polyclonal anti-GAD-65/67 (Merk Millipore, Billerica, MA, USA; code: #ABN904; 1:100) or anti-αSyn (Sigma Aldrich; code: #SAB4502828; 1:50) or anti-HSP70 (Cell Signaling Technology, Danvers, MA, USA; code: #4872; 1:50) or anti-p62 (Abcam Cambridge, UK; code: #ab109012; 1:50). Afterwards, slices were incubated for 60 min at RT with a mixture containing the following secondary antibodies: Alexa-Fluor-488 conjugated goat anti-mouse IgG (Life Technologies; code: #A10667, 1:50) and goat biotinylated anti-rabbit IgG (Vector Laboratories; code: #BA-1000, 1:200). Finally, slices were incubated for 60 min at RT with Alexa-Fluor-568 conjugated streptavidin (Life Technologies; code: #S11226, 1:200).

For double fluorescence analysis for TH and p-cJun, additional series of deparaffinized sections were subjected to antigen retrieval (10 mM, pH 6.0 citrate buffer heated in a microwave for 20 min), permeabilization in triton X-100 (0.1% in PBS for 15 min at RT), and non-specific binding sites blockade with a mixture containing 10% NGS, 10% normal donkey serum (NDS, Sigma Aldrich; code: #D9663) in 0.1% triton X-100 in PBS for 2 h at RT. After that, sections were incubated overnight at 4 °C with a mixture containing the following primary antibodies: mouse monoclonal anti-TH (Sigma Aldrich; code: #T1299, 1:100) and a goat polyclonal anti-p-cJun (Santa Cruz, Biotechnology, Dallas, TX, USA; code: sc-7981; 1:50). Afterwards, slices were incubated for 60 min at RT with a mixture containing the following secondary antibodies: horse biotinylated anti-mouse IgG (Vector Laboratories; code: #BA-2000, 1:200) and a Cy3 conjugated donkey anti-goat IgG (Jackson ImmunoResearch Europe Ltd., Trowbridge, Wilts, UK; code: #705-166-147, 1:200) and. Finally, slices were incubated for 60 min at RT with Alexa-Fluor-488 conjugated streptavidin (Life Technologies; code: #S32354, 1:200).

A final series of deparaffinized sections were used for double florescence analysis for TH immunostaining and the dihydroethidium (DHE) assay for analysis of superoxide production. To do this, sections were subjected to antigen retrieval (10 mM, pH 6.0 citrate buffer heated in a microwave for 20 min), permeabilization in triton X-100 (0.1% in PBS for 15 min at RT), and non-specific binding sites blockade (10% NGS, 0.1% triton X-100 in PBS for 2 h at RT). After that, sections were incubated overnight at 4 °C with a mouse monoclonal anti-TH (Sigma Aldrich; code: #T1299, 1:100) and then for 60 min at RT with an Alexa-Fluor-488 conjugated goat anti-mouse IgG (Life Technologies, code: A10667, 1:50). Finally, slices were subjected to incubation with DHE (Sigma Aldrich, code: #D7008-10 MG; 2 μM, for 30 min at 37 °C). 

### 4.7. TH-Positive Cell Counting in A1/C1, A2/C2 and AP Medullary Catecholamine Nuclei 

TH-positive cell density was assessed within dissectors of known dimension (900 μm^2^) randomly positioned by the software Image Pro Plus 6.2 within the area of interest (AOI) drawn by the operator at low magnification (2.5×). Inside each dissector, the TH-positive cells were detected and counted at high magnification (100×). Cell density was expressed as number per mm^2^.

### 4.8. Fluro-Jade B Staining in the RVLM/CVLM 

The presence of degenerating neurons in the RVLM/CVLM of mice subjected to METH treatment was assessed across the time windows between the first and the last injection by the Fluoro-Jade B staining (Merk Millipore; code: #AG310), as previously described [87,88]. Briefly, deparaffinized sections (10 m) were incubated for 30 min at RT in a 0.1% (*v*/*v*) acetic acid solution containing 0.001% (*w*/*v*) Fluoro-Jade B.

### 4.9. Immunohistochemical Analysis in Mesenteric Arteries 

Mesenteric arteries isolated from saline- or METH-treated mice were fixed in 4% paraformaldehyde for 10 min at 4 °C, and then subjected to blockade by incubation with 10% normal horse serum, 10% normal donkey serum and 0.1% triton X-100 in PBS for 2 h at RT. After that, sections were incubated overnight at 4 °C with a mixture containing a mouse monoclonal anti-TH antibody (SigmaAldrich; code: #T1299, 1:100) and a sheep polyclonal anti-Von Willebrand factor (vWF) antibody (Abcam; code: ab11713, 1:100). Afterwards, slices were incubated for 60 min at RT with a mixture containing a secondary biotinylated anti-mouse IgG made in horse (Vector Laboratories; code: #BA-2000) and a fluorescein conjugated donkey anti-sheep IgG (Vector Laboratories; code: FI-6000). Finally, slices were incubated for 60 min at RT with Alexa-Fluor-568 conjugated streptavidin (Life Technologies; code: S11226, 1:200). 

### 4.10. Statistical Analysis

Statistical analysis was performed by using GraphPad Prism Software, version 8.0.1. Normal distribution was assessed by the D’Agostino and Pearson test. For data showing a normal distribution, we used the unpaired two-tailed Student’s *t*-test for 2-group comparison (Figure 1C–E,C’–E’, Figure 3B, Figure 4B and Figure 5A,B) or a two-way ANOVA for repeated measures with Bonferroni’s multiple comparison test for comparison across multiple groups (Figure 1B). When data did not follow a normal distribution, or for n ≤ 6 per group, data were analyzed with the two-tailed Mann–Whitney test for 2-group comparison (Figure 1E, Figure 2A,B,D, Figure 3F, Figure 4F, Figure 8A’,B’, Figure 9A’,B’, Figure 10A’,B’ and Figure 11A’,B’). For the correlation analysis, we used the Pearson correlation test (Figure 2C,D and Figure 3C,D). Data are shown as mean ± standard error of the mean (S.E.M.). *p*-values < 0.05 were considered statistically significant.

## 5. Conclusions

This study highlighted that a reduction in the number of catecholamine neurons in A1/C1 is the neuroanatomical correlate of the METH-induced SBP sensitization in mice subjected to repeated systemic dosing with the drug.

METH-induced reduction in TH-positive cells in the CVLM/RVLM is not dependent on neurodegenerative and/or neurotoxic processes but occurs as a result of phenotypic modification events associated with a reduced intracellular free radical formation and an upregulation of the p62 autophagic factor.

## Data Availability

The data of the current study are available from the corresponding author upon reasonable request.
